# Does the Belgian diabetes type 2 care trajectory improve quality of care for diabetes patients?

**DOI:** 10.1186/s13690-015-0080-1

**Published:** 2015-07-13

**Authors:** Viviane F. A. Van Casteren, Nathalie H. E. Bossuyt, Sarah J. S. Moreels, Geert Goderis, Katrien Vanthomme, Johan Wens, Etienne W De Clercq

**Affiliations:** Scientific Institute of Public Health, Operational Direction Public Health and Surveillance, J. Wytsmanstreet 14, 1050 Brussels, Belgium; Katholieke Universiteit Leuven - Academisch Centrum voor Huisartsgeneeskunde, Kapucijnevoer 33 Blok J Bus 7001, 3000 Leuven, Belgium; UZ Leuven - MIR (Management Informatie Rapportering, Herestraat 49, 3000 Leuven, Belgium; Vrije Universiteit Brussel, Demografie, Pleinlaan 2, 1050 Brussel, Belgium; Universiteit Antwerpen, Academisch Centrum voor Huisartsgeneeskunde, Campus 3 Eiken, Universiteitsplein 1, 2610 Wilrijk, Belgium; Université Catholique de Louvain, Institut de Recherche Santé et Société, Clos Chapelle aux Champs 30, 1200 Brussels, Belgium

**Keywords:** Type 2 diabetes mellitus, Quality of care, Primary care, Chronic disease management

## Abstract

**Background:**

The Belgian care trajectory (CT) for diabetes mellitus type 2 (T2DM), implemented in September 2009, aims at providing integrated, evidence-based, multidisciplinary patient- centred care, based on the chronic care model.

The research project ACHIL (Ambulatory Care Health Information Laboratory) studied the adherence of CT patients, in the early phases of CT programme implementation, with CT obligations, their uptake of incentives for self-management, whether the CT programme was targeting the appropriate group of patients, how care processes for these patients evolved over time and whether CT start led to better quality in the processes and outcomes of care.

**Methods:**

This observational study took place in the period 2006–2011 and covered T2DM patients who started a CT between 01/09/2009 and 31/12/2011.

Four data sources were used: outcome data, from electronic patient records (EPRs) on all CT patients, provided by general practitioners (GPs); reimbursement process data on all CT patients and clinically comparable patients; and data from a sample of CT patients and clinically comparable patients from an EPR-based regional GP network and a paper-based national GP network, respectively.

Through multilevel analysis of cross-sectional and longitudinal data, the effect of CT inclusion on processes and outcome was estimated, controlling for potential confounders.

**Results:**

By the end of 2011, data on 18,250 CT patients had been collected. Approximately 50 % of these CT patients had received reimbursement for a glucometer and nearly 60 % had had at least one encounter with a diabetes educator. The CT programme recruited T2DM patients who had been difficult to control in the past. In the years prior to CT start, there had been a gradual improvement in the follow up of these patients. Moreover, compared to non-CT patients, the proportion of CT patients adhering to the recommended frequency for monitoring of parameters, such as HbA1c, increased significantly around CT start. Some data sources, albeit not all, suggested there had been an improvement in certain outcomes, such as HbA1c, after CT inclusion.

**Conclusions:**

According to this study, CT enrolment is associated with better quality of care processes compared to non-CT patients. This improvement was found in several of the data sources used in this study. However, results on outcome parameters remain inconclusive.

## Background

The Belgian health care system is based on a compulsory insurance system, which covers 99 % of the population for a wide range of health conditions. The health system focuses mainly on acute diseases and hospital care. Fee-for-service payments apply to most ambulatory and medical services, delivered by self-employed health professionals. Patients are free to choose their health care professional. General practitioners (GPs) have no formal gate-keeping function and patients have direct access to specialists and emergency services. Nevertheless, the national Health Interview Survey (HIS) of 2013 showed that a large majority of the population (94 %) has a regular GP, and that the mean number of contacts per year is 3.9 (self-reported by the patient) [1]. Moreover, a number of decisions were taken in past years to reassess and strengthen primary care. Among these, the Global Medical File (GMF) was established in 1999 to increase the availability of medical, social and administrative patient information and access to such information. The GP holds the GMF with the patient’s consent and shares relevant information with other providers responsible for the patient. Only one GP can hold the patient’s file.

Primary care is dominated by single-handed, self-employed practices for general practice, physiotherapy, speech therapy and so on, with only a limited number of integrated primary care centres. The number of such practices is growing, although there is still only a small minority of people affiliated to them.

As in most other European countries, the Belgian health care system is struggling with changing health care needs. Higher life expectancy is associated with an increasing prevalence of chronic diseases such as diabetes mellitus type 2 (T2DM), which has a growing impact on healthcare delivery and costs.

Up to 2009, the main government regulation in diabetes care was the so-called diabetes convention “786”. Since 1988, Belgian multidisciplinary hospital-based diabetic centres (over 100) can conclude a diabetes convention with the National Institute for Health and Disability Insurance (NIHDI). This convention aims at providing a self-regulation programme for people with diabetes. Initially, this convention targeted only type 1 diabetes mellitus (T1DM) and later on, the target public also included the growing number of patients with T2DM with at least 2 insulin injections a day. When the latter adhere to such a diabetic centre, they are followed up by a multidisciplinary diabetes team led by an endocrinologist. They receive reimbursement of diabetes education and self-monitoring materials and get access to specialised advice, e.g. podiatry and cardiology. According to the number of insulin injections a day and glycaemic self-control measurements, various subcategories of the diabetes convention exist, e.g. convention 3A concerns patients with two or more insulin injections a day and more than 30 glycaemic measurements a month. In 2001 a quality improvement initiative, called IQED and run by the Scientific Institute of Public Health, was installed [2, 3]. Since 2005 a diabetic foot convention exists between the NIHDI and over 30 diabetic multidisciplinary foot clinics, organised in larger hospitals, focusing on prevention and treatment of complex diabetic foot problems. A quality improvement initiative, the so called IQED Foot project is linked to this convention [4].

With the growing number of patients with T2DM, including those on insulin therapy, the diabetes convention more and more suffered from its historical weaknesses. First, the programme only targets diabetes patients with insulin therapy. All other diabetics, according to the HIS 2013 approximately 72 % of the diabetic population [5], were excluded from this care programme and had to make considerable contributions for dietetic and podiatric services and self-monitoring materials. Even a subpopulation of insulin-treated diabetes patients, those on one daily injection were excluded from this care programme as well as from the reimbursement of the necessary blood glucose monitoring material.

Further on, the diabetes convention also resulted in most diabetes patients on insulin being treated in secondary care, while many of those patients (especially those who need 2 doses of insulin a day) could be cared in primary care with the pivotal role of the GP. Moreover, insulin therapy being monopolized in diabetes centres, GPs risked to lose the necessary competences to initiate and follow-up insulin therapy. On the other hand, with the growing number of diabetes patients, the convention centres risked to be overwhelmed.

For all these reasons, there was an urgent need to reorganise the managed care for people with diabetes. Within this new system the diabetes convention could focus on diabetics with complex insulin schemes or severe complications.

However, Goderis et al. found in 2006 a better overall metabolic control in T2DM patients followed up in diabetic convention centres (with more structured care compared to primary care), compared to diabetics followed up in primary care [6].

On the other hand, Goderis et al. illustrated that a multifaceted diabetes programme in general practice with various interventions, such as a clear treatment protocol, postgraduate education of GPs, case-coaching by an endocrinologist, benchmarking feedback and possibility of patient’s referral for diabetes education free of charge, substantially improved quality of care and major diabetes-related patient outcomes [7].

In 2006, the Belgian Health Care Knowledge Centre (KCE) recommended a new organisation of the care for the rapidly increasing T2DM patient population with a shift from symptom oriented treatment to proactive integrated and patient oriented care. This new organisation of care includes, for example, involvement of a multidisciplinary team, empowerment of the patient, patient education, a central coordinating role for the GP, a possible coaching role for the endocrinologist, and a quality of care monitoring system [8].

The reflection on the need to change the present health care system for T2DM and by extension for other chronic diseases, was not only made in Belgium. In recent years, new chronic care programmes were implemented and evaluated in many other countries in and outside Europe [9–20]. Most approaches tend to be disease-specific, with T2DM most typically targeted. A common finding in the evaluation is that the programmes are associated with better diabetes care processes, but conclusions about their impact on outcomes such as clinical parameters (e.g. blood pressure, blood lipids, HbA1c), hospital admissions and mortality vary from one study to another and are not conclusive.

All these care programmes are to a large extent inspired by Wagner’s chronic care model (CCM).

For more than a decade, this CCM has been used to transform daily care for patients with chronic illnesses from an acute, reactive approach to a proactive, planned approach [21–25].

In Belgium, the care trajectory (CT) for T2DM was introduced by the NIHDI in 2009. This CT approach can be considered as the first phase of implementation of Wagner’s CCM in Belgium. First phase meaning that the focus is on a specific disease and even on a limited subset of T2DM patients and not on chronic diseases in general. The Belgian T2DM-CT aims to improve delivery of care and health outcomes by reinforcing proactively planned, integrated, evidence-based, multidisciplinary care for empowered patients [26]. It is focusing on patients in an earlier phase of their disease, in contrast to diabetes convention patients, who are often in a more advanced stage of diabetes. CT-eligible patients include T2DM patients on one or two insulin injections or GLP-analogues and those experiencing insufficient regulation with maximum oral antidiabetic treatment excluding pregnant women and T1DM patients [26].

The CT is defined by a four-year renewable contract between patient, GP and specialist. This contract aims to support the interaction between the three parties, each of whom has to respect certain rules in order to receive incentives. For example, after signing a CT contract, a patient is reimbursed in full for encounters with his or her GP and specialist [26]. He or she receives material for self-monitoring, has improved access to a diabetes educator/dietician and podiatrist, and receives partial reimbursement for these visits. On the other hand, the CT contract also stipulates that the patient must have at least two encounters a year with his or her GP and one encounter a year with the specialist.

Within the multidisciplinary care environment, the GP acts as the coordinator of the medical management according to an individualised care plan. The specialist’s role is to keep the GP’s knowledge up-to-date and coach him or her in relation to individual patients. In order to facilitate multidisciplinary care, new local structures, known as “local multidisciplinary networks” (LMN), were set up with the financial support of the NIHDI. In these networks, a new care manager function, the care trajectory promoter, was introduced [26].

Before including the patient in a T2DM-CT programme, the GP can eventually start a diabetes education and self-management programme, in which only the GP provides diabetes education and the patient receives free material for self-monitoring of blood glucose [26]. There is no multidisciplinary approach in this latter care programme. Inclusion criteria for the patient are being treated with either injectable incretinemimetics or one insuline injection per day. If after one year of inclusion in this programme the patient’s HbA1c level is not < 7.5 %, the GP proposes the patient to join the T2DM-CT care programme.

There is some overlap between the diabetes convention 3A and the T2DM-CT for patients on two insulin injections per day. Convention 3A patients can stay in the convention or move to a T2DM-CT, but once in a T2DM-CT, they are not allowed to switch to the convention 3A [26].

The research project ACHIL (Ambulatory Care Health Information Laboratory) assessed the adherence of CT patients, in the early phases of CT programme implementation, to the CT rules; their uptake of incentives for self-management; whether the CT programme was targeting the appropriate group of patients; how care processes for these patients evolved over time; and whether inclusion in the CT led to better quality in the processes and outcomes of care. Both crude effectiveness (proportion of patients meeting a target value) and comparative effectiveness (comparison with a control group or over time) were assessed. With regard to the latter, various groups of T2DM patients were used: T2DM patients in a care programme on diabetes education and self-management, diabetes convention 3A patients on two insulin injections a day, CT-eligible T2DM patients not included in a CT and T2DM patients in no specifically dedicated care programme.

This article describes an extract of the ACHIL project’s T2DM-CT-related research objectives, the approach to meeting these objectives, and some general results from the early phases of T2DM-CT implementation. More details on the methodology and some specific results are reported elsewhere [27–31].

## Methods

### Research domains, research questions and quality-related parameters

Table 1 gives an overview of the research domains and (quality-related) parameters considered in this article, together with the studied target frequency of measurement and target outcome value. The research questions on quality of care of diabetes were selected in accordance to a 2006 report of the Belgian KCE on quality of T2DM care [8] and the follow-up plan for the care trajectory diabetes, developed by the National Council for Quality Promotion and based upon the national recommendation for good medical practice [32]. The KCE report classified relevant guidelines and the resulting evidence based quality indicators (at micro level) from international reference literature within major areas of diabetes care. Appropriate target values to these quality indicators have been assigned. The study method was based on a study evaluating the diabetes convention carried out by the Intermutualistic Agency [33] and on a study evaluating a diabetes care quality improvement programme for general practitioners that was carried out by the KU Leuven and the Universiteit Antwerpen [7, 34].

**Table 1:** Research domains and parameters under study for type 2 diabetes care trajectory (T2DM-CT). 1 September 2009 – 31 December 2011

Research domains	Parameters studied	Target frequency/target value
Recruitment of CT patients	Number of enrolled patients	
Mandatory parameters for all T2DM-CT patients	HbA1c	Freq: every 3 months
		Target value: <7 %
	LDL cholesterol	Freq: every 3 months
		Target value: <100 mg/dl
	Blood pressure	Freq: every 3 months
		Target value: <130/80 mmHg
	BMI	Freq: every 3 months
		Target value: <25 kg/m2
Disease review	Number of encounters with selected care providers	2 encounters/year with GP
		1 encounter/year with internist
	Prescription of a glucometer	Number T2DM-CT patients with prescription of glucometer
Primary revention	Flu vaccination	Annual flu vaccination
Complications/secundary and tertiary prevention	Diabetes education	At least 1 consultation with diabetes educator/dietician
	Renal function	Freq: Serum creatinine 1×/year
	Statin use	All T2DM, except those without other cardiovascular risk factors
	Ophthalmoscopy	Freq: 1×/year

### Data sources

Four data sources were selected in order to answer the research questions. Detailed information about the four data sources is reported elsewhere [35].

### Central pillar

This data source was the result of the compulsory recording of four parameters (HbA1c, LDL cholesterol, blood pressure and BMI), with at least one value per parameter, by Belgian GPs for all T2DM-CT patients (see Table 1). Data were entered by GPs using a secure web application, either manually or with the help of automatic data extraction from electronic patient records (EPRs), encrypted and sent to the Scientific Institute of Public Health [28, 29, 36]. The data collected cover the period from 1 June 2008 to 31 December 2011, enabling an observational retrospective cohort study.

### IMA pillar

This pillar drew on the Intermutualistic Agency (IMA)’s administrative database of national compulsory health insurance data which includes data from all seven Belgian not-for-profit health insurance providers [37]. This database contains information on all reimbursed medications, medical and paramedical interventions and hospital admissions as well as socio-demographic data on all Belgian citizens who have health insurance. Data relevant to our research questions were collected covering the period between 1 January 2006 and 31 December 2010, enabling an observational retrospective cohort study. Data discussed in this article concerned frequency of measurement for HbA1c, LDL cholesterol, serum creatinine, number of encounters with selected care providers, prescription of glucometer, flu vaccination, consultations with diabetes educator and statin use.

### Intego registration network

The Intego network, operational since 1997, is an EPR-based network of 54 voluntarily participating GP practices in Flanders, the northern region of the country, which all use the same EPR software [38]. The network is coordinated by the Academic Centre for General Practice at the Katholieke Universiteit Leuven. The network covers approximately 1.95 % of the Flemish population. Information was extracted on all encounters and included individual demographic and administrative data, clinical and laboratory parameters, vaccinations and prescriptions.

The data used in this study covered the period between 1 January 2006 and 31 December 2011, enabling an observational retrospective cohort study. Data concerned frequency of measurement and values for HbA1c, LDL cholesterol, blood pressure, BMI, serum creatinine, flu vaccination and statin use.

### Sentinel network of general practices

The Belgian Sentinel Network of General Practices (SGP), operational since 1979, is a paper-based nationwide network which collects data from approximately 140 voluntarily participating practices on a limited set of parameters related to specific health problems [39]. The network covers about 1.5 % of the total Belgian population. Type 2 diabetes was among the themes recorded in 2010. Cross-sectional data concerned the three most recent values for HbA1c, blood pressure and weight, and the most recent value for height and LDL cholesterol.

### Data analysis

Logistic and continuous multilevel analyses were carried out to study the effect of inclusion in the CT on achieving a care process or a health outcome target, controlling for potential confounders.

Triangulation of the four data sources was carried out [40], as each source has its own strengths and weaknesses [35].

This article examines data from the early phases of CT implementation and the years preceding the CT start. The data used covered the period from 2006 to 2011.

## Results

### Number of T2DM-CT patients and their basic characteristics

Table 2 describes the characteristics of the registered T2DM-CT cases by data source. The age and sex distributions and geographical spread of the four data sources were comparable.

**Table 2:** Characteristics of type 2 diabetes care trajectory patients by data source. 1 September 2009 – 31 December 2011

	Central pillar	IMA pillar	Intego	SGP
Enrolment period	1 Sept 2009 – 31 Dec 2011	1 Sept 2009 – 30 June 2010	1 Sept 2009 – 31 Dec 2011	Cross-sectional data: status on 31 Aug 2011
Number of patients	18250	8528	271	95
Median study time	15 months	11 months	13 months^a^	not applicable
Ratio of men:women	1.08	1.03	1.08	0.79
Median age(group)	68y	65–69y	69 y^a^	67y
Geographic region				
Flanders	83 %	89 %	100 %	80 %
Wallonia	13 %	9 %	0 %	13 %
Brussels Region	4 %	3 %	0 %	7 %

^a^Mean instead of median

A considerable number of patients were already enrolled in a CT in this early phase of CT implementation, as illustrated by the IMA and central pillar data.

### Patients’ adherence with T2DM-CT obligations

According to the CT care programme rules, every CT patient must have at least two encounters a year with his or her GP and one encounter a year with the internist. The IMA data indicate that 97 % (95 % CI: 96–97) of the CT patients had at least two encounters with their GP and 95 % (95 % CI: 94–95) had at least one encounter with the specialist in the year 2010.

### Patients’ uptake of measures for T2DM-CT self-care

The CT care programme facilitates self-monitoring of glucose through prescription and reimbursement of a glucometer. According to the IMA data, 50 % (95 % CI: 49–51) of the CT patients used this incentive in the period September 2009 – December 2010. The CT care programme also facilitates diabetes education by reimbursing consultations. According to the IMA data, 58 % (95 % CI: 56–59) of the CT patients had at least one consultation with a diabetes educator or dietician in the period September 2009 – December 2010.

### Process of care for T2DM-CT patients prior, around and after CT start

We observed a gradual improvement in the follow-up of CT patients’ biological parameters, such as HbA1c and LDL cholesterol, and clinical parameters, such as blood pressure measurement, in the years before CT start (data not shown). However, the target frequency with which specific care processes should be carried out, had still not been attained for a considerable proportion of patients. This was the case, for instance, in the follow-up of ophthalmoscopy and flu vaccination, where according to the IMA data of 2008 only 33 % (95 % CI: 32–34) of the patients had an ophthalmoscopy and 66 % (95 % CI: 65–67) have been vaccinated against flu.

On top of the improvement observed in the care processes during the years before CT start, we also saw a significant increase, around the start of the CT, in the proportion of CT patients receiving adequate care (Table 3).

**Table 3:** Process of care parameters around the type 2 diabetes mellitus (T2DM) care trajectory (CT) start: degree of improvement in follow-up per trimester around individual CT start dates. IMA data, 2010, ACHIL project, Belgium

Process parameter	Frequency of measurement/year	Improvement 3 months before CT start
		Odds ratio (per trimester)
HbA1c	≥3	1.34*
LDL cholesterol	≥1	1.41*
Serum creatinine	≥1	1.42*
Ophtalmoscopy	≥1	1.15*
Statines	≥1 prescription	1.15*
Flu vaccination	≥1 vaccination	0.73* (per year)

A value above 1 indicates an improvement in time per trimester. The symbol* after a value indicates that it is statistically significant, with *p* < 0.05

After this significant increase, the process parameters decreased slightly, but nevertheless remained at a higher level than prior to CT start.

**Fig. 1:**
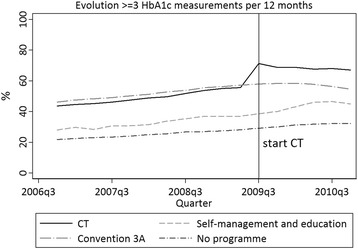
Proportion of diabetes type 2 care trajectory (CT) and non-CT patients with > =3 HbA1c measurements around the CT start, IMA pillar, 2006–2010. Proportion of diabetes type 2 (T2DM) care trajectory (CT) patients with > = 3 HbA1c measures around CT start, in comparison with T2DM patients on a diabetes convention 3A care programme (two insulin injections a day, treated in specialised diabetic centres), with T2DM patients in a care programme on diabetes education and self-management and with T2DM patients in no dedicated care programme

The significant increase in frequency of T2DM-CT patient follow-up, we observed around the start of the CT, was far less notable among other T2DM patients (Fig. 1).

### Outcome of care for T2DM-patients prior and after CT start

**Fig. 2:**
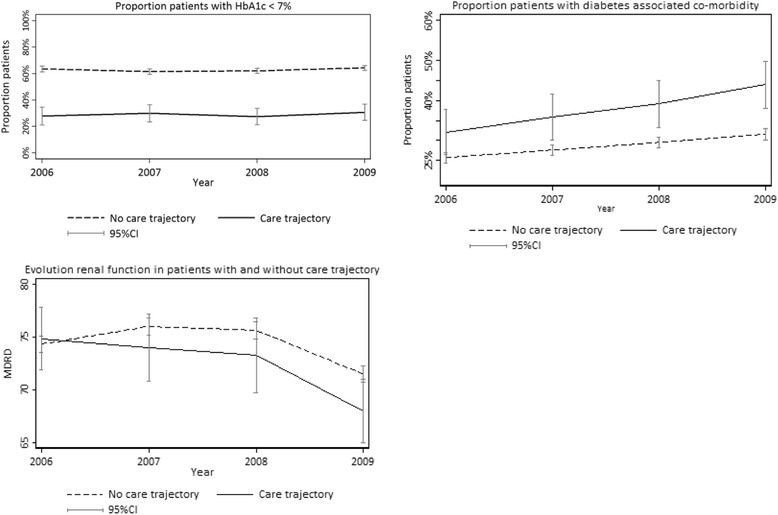
Clinical status prior to care trajectory (CT) start in diabetes type 2 CT and non-CT patients, Intego network, 2006–2009. Proportion of patients included and eligible patients not included in type 2 diabetes mellitus care trajectory, with HbA1c < 7 %, diabetes-associated co-morbidity and renal function progression, prior to CT start

The T2DM-CT care programme recruited high-needs patients, who, according to the Intego network, prior to the start of the CT, had higher HbA1c levels, more rapidly declining renal function, and more diabetes-associated co-morbidity compared to non-CT diabetic patients (Fig. 2).

CT patients were difficult to control in the years preceding the CT start. According to the SGP network, only 38 % (95 % CI: 28–48) of the CT patients had prior to the CT start a HbA1c value <7 %, 11 % (95 % CI: 5–18) had a blood pressure < 130/80 mmHg, and 8 % (95 % CI: 2–14) had a BMI <25 kg/m^2^. The proportion of CT diabetics with an LDL cholesterol <100 mg/dl, on the other hand, was considerable (61 %, 95 % CI: 41–81). However, about two thirds of these patients were taking statins.

**Fig. 3:**
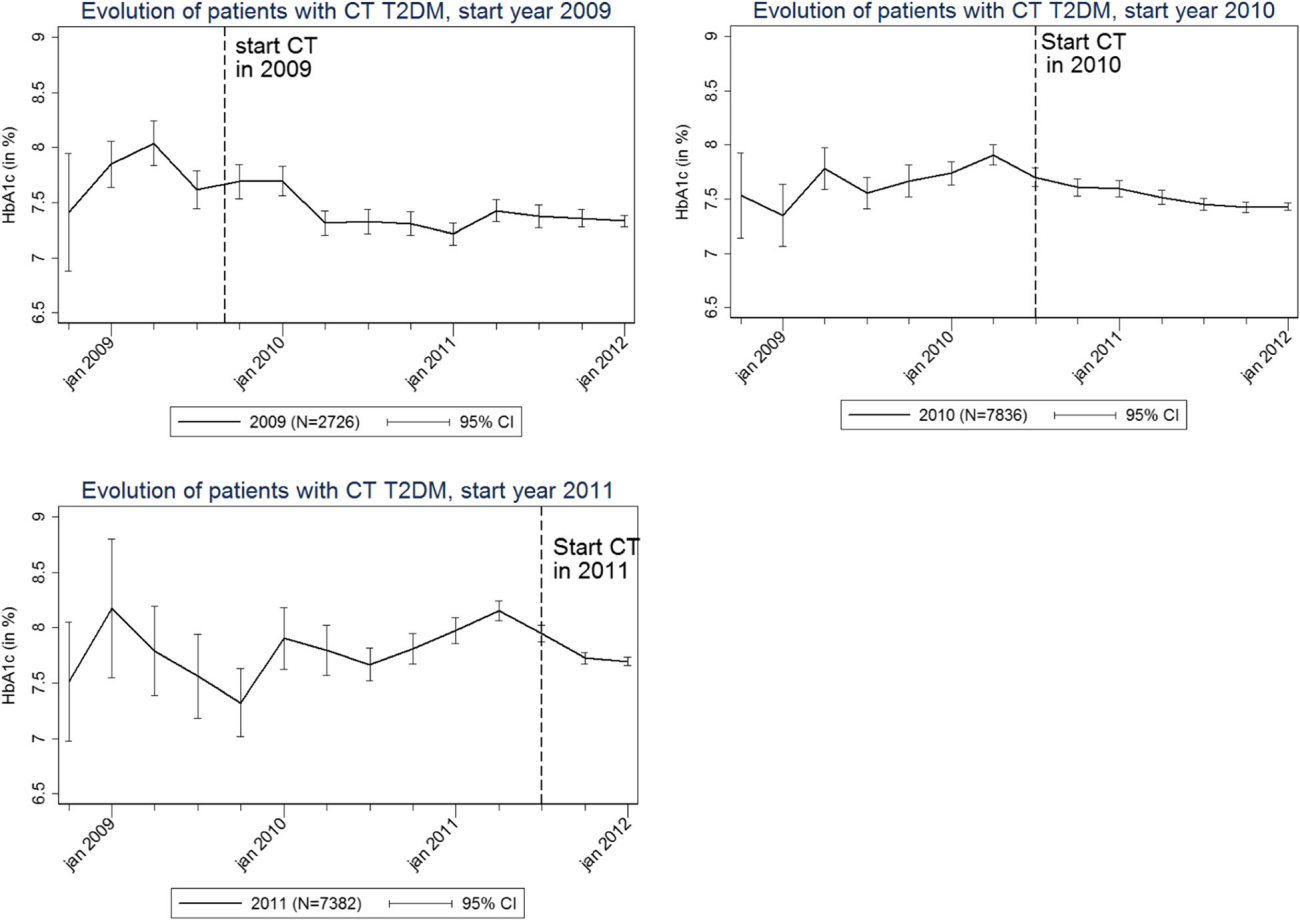
HbA1c levels in diabetes type 2 care trajectory (CT) patients by year of CT start, central pillar data 2008–2011. HbA1c levels in diabetes type 2 care trajectory (CT) patients after CT start, for the cohort of CT patients who started the CT in 2009, 2010 and 2011

The central pillar data showed a significant decrease, after CT start, in HbA1c among three cohorts of T2DM-CT patients, with CT start in 2009, 2010 and 2011, respectively (Fig. 3). This finding was confirmed by the Intego dataset [30]. According to the central pillar data, T2DM-CT patients also experienced a significant decrease in LDL cholesterol, blood pressure and BMI after CT start (data not shown). However, these findings were not confirmed by the Intego dataset.

## Discussion

This first phase of the ACHIL project aimed at studying the impact of the T2DM-CT care programme on quality of care, in terms of processes and outcomes, in the early period of the CT care programme implementation.

The focus was on quality of care in its narrowest sense (quality of technical care) in which practitioners’ performance is measured by looking at its effectiveness to realize the achievable improvements in health status that have been made possible by the current science and technology of health care [41]. The assessment of quality of care in this study was limited to two levels: process (what is actually done in giving and receiving care), and outcome (effects of care on the health status of patients and populations). Other dimensions of quality of care, such as efficiency, patient and care providers satisfaction, were not dealt with in the ACHIL project.

This study revealed that a considerable number of T2DM patients had already been enrolled in the early phase of the CT care programme implementation. More than 50 % of the CT patients we studied were reimbursed for a glucometer. This care programme was effective in recruiting high-needs patients, who were difficult to supervise and had already undergone a more intensive follow-up prior to CT start. The vast majority of CT patients had the required number of encounters with their GP and the internist and more than 50 % had at least one encounter a year with a diabetes educator or dietician. The CT care programme also proved to be effective in improving the follow-up of the patients enrolled, compared to both their own follow-up in the past and the follow-up of other diabetic patients. Despite the emergence of a number of positive trends, however, no conclusive results have yet been obtained in relation to health outcome parameters.

By 31 December 2011, the central pillar data-set had captured data on 18,250 T2DM-CT patients. Producing a reliable estimation of the number of patients eligible for a CT was impossible, however, due to the lack of data on the occurrence of the inclusion and exclusion criteria for a CT within the general population and the difficulty of obtaining reliable estimates of T2DM prevalence in Belgium. Nevertheless, the number of CT patients included in this study was considerable, and had increased to 46,561 by September 2014, according to recent NIHDI figures (NIHDI, personal communication).

According to the data provided by the Belgian Sentinel Network of General Practices (SGP), 16 % (95 % CI: 13–19) of all CT-eligible patients recorded by the network had been included in a CT by the end of the surveillance period in August 2011. The younger the CT-eligible patient, the more likely he or she was to be included in a CT. Patients living in Flanders (the northern region of the country) were more likely to have been included than were patients living in Wallonia (the southern region of the country). Motivated patients with specific plans to change their diet were also more likely to have been included in a CT. The most frequently reported reasons for non-inclusion, as mentioned by the GP, were the early timing of this study (inclusion was to take place in the near future) and inclusion in the diabetes convention 3A programme [31].

Despite the reimbursement facilities, consultations with diabetes educators did not occur as frequently as expected in this early phase of CT implementation. Several elements could have contributed to this underuse, such as the considerable administrative burden placed on GPs when making referrals to educators, the low financial compensation provided to educators, the insufficient amount of time allotted per education session, the lack of an accreditation system for diabetes educators, or patient factors other than financial incentives (e.g., perceived seriousness of the illness, external locus of control, low perceived self-efficacy, lack of social support,…).

The increased follow-up of various process parameters observed among T2DM-CT patients can be considered as an improvement in quality of care, as defined in evidence-based guidelines. For several of these process parameters, however, there is still considerable room for improvement. This is the case, for instance, for the yearly ophthalmoscopy exam, for which under 50 % of the CT patients we studied conformed to the recommended frequency of follow-up.

Despite the emergence of a number of positive trends in health outcomes, no conclusive results have been obtained to date: a decrease in HbA1c, LDL cholesterol or blood pressure among T2DM-CT patients cannot necessarily be attributed to the CT programme. Additionally, an improvement in outcomes found in one data source was not always confirmed by another source. Any improvements in outcome were also rather small and it seems premature to consider them obvious improvements in a patient’s health status (with slower disease progression as a result). Further research is needed to assess the trends in health outcomes due to the T2DM-CT.

The findings of this study corroborate with the evaluation results of other care programmes for chronic disease management in various countries in and outside Europe [9–20].

A criticism of the Belgian diabetes CT care programme is its focus on patients who already have advanced diabetes. While our results show that targeting this group of diabetics is justified, it is also known that the greatest benefit in terms of health and costs could be achieved by treating early-diagnosed patients well [42, 43]. The need to extend this diabetes care programme has been recognised by the Belgian public health authorities and is presently under discussion. A second issue to be tackled is the present co-existence of the T2DM-CT and the diabetes convention 3A care programme, which to some extend target the same patients.

Another criticism of the Belgian CT care programme is the lack of a holistic approach. To date, two different CT programmes exist, one for T2DM and another for chronic renal failure. However, co-morbidity has not been taken into account in this approach, although the vast majority of elderly people have at least two chronic disorders. This criticism has also been recognised by the health authorities and therefore, the NIHDI recently established a working group on T2DM and multimorbidity.

This study has a number of limitations that should be acknowledged. The Belgian CT care programme is an example of a complex intervention, consisting of various elements: a contract, patient education measures, reimbursement of various consultations and self-control material, multidisciplinary local structures, and so on. The effectiveness studied here, concerns the CT care programme as a whole and not the individual elements.

The ACHIL project only focused on the effectiveness of the T2DM-CT programme in terms of quality of care processes and outcome. The CT being a complex intervention, it is likely that the participation of the patients and the effectiveness of the program not only depends on the program itself, but also, among other factors, on the patients’ level of (health) literacy. The role of health literacy as a moderator of diabetes treatment effectiveness has been documented in the literature [44]. Besides, the same complexity could be a reason for the implementation fidelity of the program to be limited. Implementation fidelity in diabetes self-management programs is also mentioned as a potential mediating factor of effectiveness [45].

Another limitation of this study is that it concerns an observational study, which has a number of shortcomings compared to a randomised controlled study. Patients who were eligible for a CT, but not enrolled, were difficult to define in the data sources and, in addition, could not serve entirely as a control group for the CT patients. Besides this, the observation period was very short: no data were included after 2011, although the CT care programme started only in the summer of 2009. In the central data pillar, automatic extraction from EPRs appeared to be more promising than manual data entry. In 2012, however, only 9 % of all CT patients’ data were extracted from the EPR and many fields remained incomplete.

An important strength of this study is the fact that it involved the first large-scale (partly EPR-based) data collection, and included all GPs and CT patients. Another strength of the study is the use of triangulation between the four data sources. The conclusions of this study are based on congruent results obtained from the various data pillars, thus enforcing the validity of the conclusions and the validity of each data source individually. The framework of four different data sources developed here can be considered as an important basis for further evaluation of the effectiveness of the Belgian CT care programme.

## Conclusions

In 2009, the NIHDI introduced care trajectories (CTs), a chronic disease management programme involving complex interventions. The evaluation of this programme’s implementation is essential for the public health authorities. An important aspect of this evaluation consists of studying the effectiveness of the CT care programme with regard to the quality of care delivered and the outcome of care for patients. This study deals with this evaluation in the early phases of its implementation.

The ACHIL study found that, in the period 2009–2011, the CT care programme was effective in recruiting high-needs patients and in improving the processes of care for those patients. To date, however, no conclusive results have been obtained regarding the impact of the care programme on patient health outcomes.

As a result of this study, we recommend confirming the improvements found in care processes; further investigating patient health status outcomes and strengthening quality improvement by means of quality circles (plan-do-check-act). In order to achieve these recommendations, a longer observation period is needed, in combination with improved automatic data extraction from EPRs, monitoring of this improvement, linkage of data from EPRs with reimbursement data, extension of compulsory parameters in the central pillar and continuation of the triangulation between multiple data sources.

## Abbreviations

ACHIL: Ambulatory Care Health Information Laboratory
BMI: Body mass index
CCM: Chronic care model
CI: Confidence interval
CT: Care trajectory
EPR: Electronic patient record
GLP-analogues: Glucagon-like peptide analogues
GMF: Global Medical File
GP: General practitioner
HIS: Health Interview Survey
IMA: Intermutualistic Agency
KCE: Belgian Health Care Knowledge Centre
LDL: Low-density lipoprotein
NIHDI: National Institute for Health and Disability Insurance
SGP: Sentinel Network of General Practices
T1DM: Type 1 diabetes mellitus
T2DM: Type 2 diabetes mellitus
